# Takayasu Arteritis: From Diagnosis to a Life-Threatening Complication

**DOI:** 10.5935/abc.20180195

**Published:** 2018-10

**Authors:** Filipa Cordeiro, Sofia Silva Carvalho, Fernando Salvador, Alberto Ferreira, J. Ilidio Moreira

**Affiliations:** Centro Hospitalar de Trás-Os-Montes e Alto Douro, Hospital de Vila Real, Portugal

**Keywords:** Takayasu Arteritis/surgery, Aortitis/physiopathology, Takayasu Arteritis/diagnostic imaging, Vasculitis.

**Figure 1:**
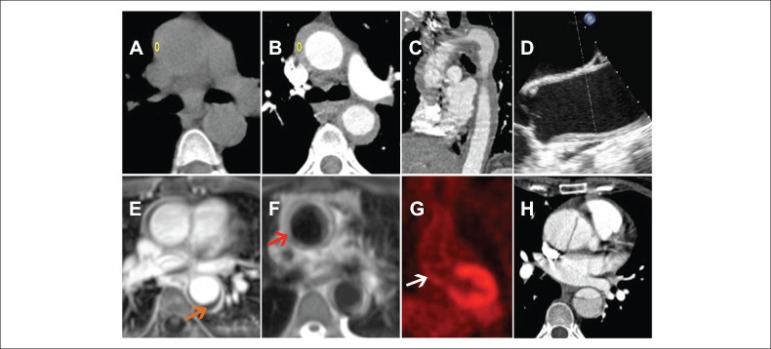
A) Non-contrast computed tomography showing low-attenuation concentric mural thickening of the thoracic and abdominal aorta (43 HU). B and C) Computed tomography angiography revealing enhancement of the mural thickening of the thoracic and abdominal aorta (73 HU). D) Transesophageal echocardiogram presenting thickening of the thoracic aorta after the Valsalva sinus. E and F) Cardiovascular magnetic resonance imaging demonstrating that mural thickening was hypointense on T1-weighted images (E, orange arrow) and hyperintense on T2-weighted images (F, red arrow), consistent with aortitis. G) Positron emission. Tomography after fifteen days of steroid therapy showing a discrete tracer uptake in the thoracic aorta (white arrow). H) Computed tomography angiography revealing type A aortic dissection six weeks after the initial diagnosis of Takayasu arteritis.

A fifty-two-year-old Caucasian woman was admitted for severe epigastric pain irradiating to the back. Physical examination and electrocardiogram were normal. Laboratory tests showed leucocytosis (11100 cells/uL) and increased levels of C-reactive protein (15.6 mg/dl). Due to the suspicion of acute aortic syndrome (AAS), she underwent computed tomography (CT), which showed a low attenuation circumferential mural thickening of the aorta (43 Hounsfield units (HU)), which enhanced (73 HU) after contrast administration (Figures 1 A-C), suggestive of aortitis.^1^ Transesophageal echocardiogram also revealed thickened thoracic aorta (Figure 1 D). Cardiovascular magnetic resonance imaging confirmed the diagnosis of aortitis and excluded intramural hematoma (mural thickening hypointense on T1-weighted images and hyperintense on T2-weighted images)^1^^,^^2^ (Figures E-F). Infectious serologies were negative.

The patient was diagnosed with Takayasu arteritis (TA) at initial inflammatory phase and initiated treatment with high-dose steroids. There was a reduction of serum inflammatory markers and aortic wall inflammation. Positron emission tomography after fifteen days of therapy showed a discrete tracer uptake in the thoracic aorta (Figure G). After six weeks of treatment, the patient initiated severe back pain. CT angiography showed type A aortic dissection (Figure H). She underwent emergent cardiac surgery, which included resection of ascending aorta, replacement with an artificial graft and obliteration of distal false lumen. Postoperative period was uneventful.

TA is a rare, large-vessel vasculitis characterized by an inflammatory phase followed by a pulseless phase.^3^^,^^4^ Multimodality imaging is useful for diagnosis, which can be challenging due to the similarities with AAS, and follow-up.^1^^,^^2^ Aortic dissection is an exceptionally rare complication.^5^
